# In Vitro Cytotoxic and Molecular Docking Studies of the Network Pharmacology Approach From Bioactive Compounds of *Coleus amboinicus* Leaves Against Lung and Breast Cancer Cells

**DOI:** 10.1155/adpp/5946648

**Published:** 2025-07-11

**Authors:** Kasta Gurning, Gian Primahana, Endang Astuti, Winarto Haryadi

**Affiliations:** ^1^Department of Chemistry, Faculty of Mathematics and Natural Sciences, Universitas Gadjah Mada, Yogyakarta 55281, Indonesia; ^2^Department of Pharmacy, Sekolah Tinggi Ilmu Kesehatan Senior Medan, Medan 20141, Indonesia; ^3^Research Center for Pharmaceutical Ingredients and Traditional Medicine, National Research and Innovation Agency (BRIN), South Tangerang 15314, Indonesia

**Keywords:** breast cancer, *Coleus amboinicus*, lung cancer, MMP-2, MTT

## Abstract

Lung cancer and breast cancer are two types of cancer that cause and contribute to the highest mortality rate in the world. The development of anticancer agents that have high efficacy and relatively low side effects continues to be developed and is the focus of research, and one of the raw materials that can be explored is active compounds sourced from natural materials, one of which is plants. This study aims to isolate active compounds from bangun-bangun (*Coleus amboinicus*, Lour.) leaves, test cytotoxicity as an antilung and breast cancer agent in vitro, and conduct molecular docking studies with a network pharmacology approach on the pathways in cancer. Research methods include extraction by the maceration method and purification by column chromatography, anticancer activity testing is carried out by the microtetrazolium (MTT) test method against lung cancer (A549), breast cancer (MCF-7), and normal cells (CV-1), and molecular docking studies are carried out with a network pharmacology approach focused on proteins in cancer pathways. The results of the active isolate (I_nH-2_) from *n*-hexane extract showed the best activity against lung cancer/A549 cells (IC_50_ 31.74 μg/mL), and the active isolate (I_EtOAc-1_) from ethyl acetate extract showed the best activity against breast cancer/MCF-7 cells (IC_50_ 80.05 μg/mL) and showed no toxicity to normal cells (CV-1). The results of the bioinformatic study are with a network pharmacology approach on the cancer pathway of bioactive compounds from each isolate target the matrix metalloproteinase-2 (MMP-2) protein. The content of bioactive compounds from *Coleus amboinicus* leaves shows the potential to be used as active agents in the treatment of lung cancer and breast cancer in the future.

## 1. Introduction

Bangun-bangun (*Coleus amboinicus*, Lour.) is a plant that is rich in nutrients and provides various pharmacological activities. Pharmacological activities include antioxidant, diuretic, analgesic, cancer prevention, antitumor, antivertigo, immunostimulant, anti-inflammatory, antifertility, hypocholesterolemia, hypotension, antimalarial, antimicrobial, antidiabetic [1–3], antitumor against Sarcoma-180 and Ehrlich ascites carcinoma, antirheumatoid arthritis, and anticancer [4]. The potential activity is supported by various types of bioactive compound content including flavonoids, monoterpenoids, diterpenoids, triterpenoids, sesquiterpenoids, phenolics, flavonoids, alcohols, and essential oil aldehyde esters [5, 6]. One of the diseases that is of serious concern to find a raw material for medicine is cancer.

Cancer is caused by high exposure to free radicals [7] and is the third largest contributor to death [8]. The deadliest type of cancer is lung cancer [9], and most will be detected at an advanced stage [10]. This disease occurs 80%–90% in the body caused by cigarette tobacco [11, 12] and can occur in both men and women, and women are more at risk than men due to exposure to cigarette smoke [13, 14]. Lung cancer is divided into non-small-cell lung cancer (NSCLC) and small-cell lung cancer (SCLC) [13, 15]. Another type of cancer that is of serious concern is breast cancer [16–18]. Breast cancer is the most common cancer in women in the world, accounting for 36% of oncology patients. Breast cancer is a malignant tumor that continues to increase in regions around the world and is highest in industrialized countries [14]. This disease is a serious health problem to find and develop treatments. The treatments commonly used so far are chemotherapy and radiation. This treatment has nonspecific drug action and major side effects and can cause drug resistance. One solution to drug development is a drug compound based on natural ingredients because the therapeutic effect will be the main source of new drug development. Therefore, this study aims to isolate active compounds from *C. amboinicus* leaves, test the cytotoxicity as an antilung and breast cancer agent in vitro and conduct molecular docking studies with a network pharmacology approach on the pathways in cancer.

## 2. Materials and Methods

### 2.1. Materials

Samples of *C. amboinicus* leaves, ethanol, n-hexane, chloroform, ethyl acetate, distilled water, silica gel 60 (0.02-0.5 mm), thin layer chromatography (TLC) silica gel 60 RP.18 F254S, chromatography column, stirring rod, separating funnel, funnel, chamber, capillary tube, 10 mL vial bottle, Whatman filter paper No. 1, and macerator. Characterization and analysis of active isolates using Barnstead electrothermal IA9300, UV-Vis lamp (254 and 366 nm), UV-Vis spectrophotometer (Shimadzu), FT-IR (KBr) spectrophotometer 8201PC (Shimadzu), gas chromatography-mass spectroscopy (GC-MS) (C2010 MSQP 2010S Shimadzu), and H and 13C-NMR (Roesy NMR Bruker Ascend 700 MHz). Cytotoxic analysis using DMSO (Merck), cisplatin (EDQM C22100000), trypsin-EDTA, 3-(4,5-dimethylthiazol-2-yl)-2,5-diphenyltetrazolium bromide (MTT), 96-well plate (Nest Brand 701001), PrestoBlue™ cell viability reagent (Thermofisher A132), ELISA microplate reader, doxorubicin, antibiotics (Sigma), RPMI-1640, PBS (Gibco 18912-014), and DMEM-H media (Gibco 11875-093).

### 2.2. Preparation and Isolation of Bioactive Compounds From *C. amboinicus* Leaves

**Figure 1:**
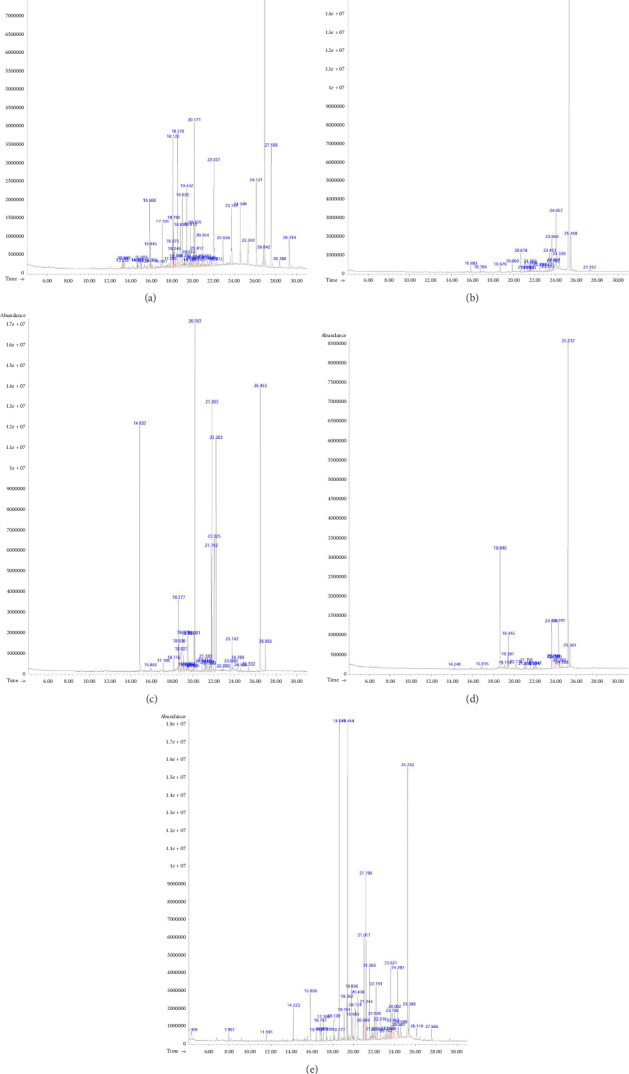
GC chromatogram of active isolates of *C. amboinicus* leaves: (a) I_nH-1_; (b) I_nH-2_; (c) I_EtOAc-1_; (d) I_EtOAc-2_; (e) I_EtOAc-3_.

The extraction and partitioning process refers to previous research [19], and a description of the research implementation is shown in Figure 1. The partitioned *n*-hexane and ethyl acetate extracts were continued for purification using gravity column chromatography. The stationary phase used silica gel, and the mobile phase used a mixture of *n*-hexane and ethyl acetate solvents with increasing polarity. The eluate that passed through the chromatography column was collected in a 10 mL vial bottle whose eluate storage volume was based on the color fluorescence produced in the stationary phase. The eluate collected in the vial bottle was evaporated at room temperature to obtain isolates in concentrated and crystalline conditions. The isolates obtained were characterized (organochemical) including color and shape, melting point, and color fluorescence produced using a TLC plate observed under UV-Vis lamp at 254 and 366 nm. Analysis of the isolates' structure was conducted using UV-Vis, FT-IR_(KBr)_, GC-MS, and ^1^H and ^13^C-NMR spectrophotometers [2, 3, 20].

### 2.3. Cytotoxicity Testing Against A549, MCF-7, and CV-1 Cancer Cells

The procedure for cytotoxicity testing against lung cancer cells (A549), breast cancer cells (MCF-7), and normal cells (CV-1) refers to previous studies [21]. Briefly, cytotoxicity testing against cancer cells was carried out using the MTT method, which began with cancer cell culture with RPMI and DMEM-H media containing 10% PBS and 50 μm/mL antibiotics incubated at 37°C with 5% CO_2_ until the growth percentage was 70%. The concentration of isolates used was 7.81, 15.63, 31.25, 62.5, 125, 250, 500, and 1000 μg/mL with DMSO solvent. Positive controls as cancer drugs include doxorubicin (lung cancer) and cisplatin (breast cancer).

### 2.4. Analysis With a Pharmacological Network Approach

#### 2.4.1. Prediction of Potential Target Proteins From Isolates

Isolates from structural analysis results from *n*-hexane and ethyl acetate extracts were predicted genes using SMILE data and then uploaded to the SwissTargetPrediction database site (https://www.swisstargetprediction.ch/). Data in CSV format were downloaded and filtered to obtain potential genes from each active isolate based on the probability score value.

#### 2.4.2. Prediction of Target Proteins From Cancer

Exploration and collection of target genes from cancer with the keywords “lung cancer” and “breast cancer” from the National Center for Biotechnology Information GeneCards disease database (https://www.genecards.org/), downloaded and filtered using genes with protein coding. Then the obtained genes that correlate with human cancer are corrected using the TTD database (https://db.idrblab.net/ttd/), OMIM (https://www.omim.org/), and DisGeNET.

#### 2.4.3. Protein–Protein Interaction (PPI) Construction of Active Isolates With Cancer

PPI from active isolate genes and target cancer disease genes (lung cancer and breast cancer) is uploaded to the Venn diagram (https://bioinfogp.cnb.csic.es/tools/venny/index.html) to obtain gene overlap between active compounds and target disease genes and then uploaded to the STRING website (https://string-db.org/) separately with the *Homo sapiens* organism settings. PPI interactions use a confidence level of 0.4 and other parameters as default. The tsv file is exported and then imported into Cytoscape 3.10.2 and then identified and analyzed using CytoHubba. Potential genes are ranked based on the number of edges, betweenness centrality, average shortest path length, clustering coefficient, eccentricity, indegree, and closeness centrality.

#### 2.4.4. Gene Ontology (GO) and Kyoto Encyclopedia of Genes and Genomes (KEGG) Analysis

GO and KEGG were analyzed comprehensively, including GO biological processes (BPs), GO molecular functions (MFs), GO cellular components (CCs), and gene functionalities. GO and KEGG analyses used the ShinyGO 0.80 database uploaded to the site https://bioinformatics.sdstate.edu/go/ with a statistical significance level of 0.05. The results of the analysis will provide important information about the main genes that have key functional roles in each pharmacological pathway influenced by the activity of active isolates against the disease being investigated. The genes that were further investigated from the activity of active isolates and became the main genes were the general cancer pathway pharmacology network genes that were aligned with potential genes from the PPI results. The main genes obtained became important proteins used for molecular docking studies.

### 2.5. Molecular Docking

Molecular docking is a rational method for exploring the interaction between bioactive compounds (ligands) and main proteins (receptors). This step is important because we get information on the prediction of the type of bond that occurs between bioactive compounds and proteins, the prediction of intermolecular patterns that provide biological activity, and their affinity energy [22, 23]. The steps taken are to optimize each bioactive compound structure with GaussView 5.0.8 to obtain an ideal 3D structure. Furthermore, charge correction is carried out using the Chimera application (Version 1.14). The primary target proteins were downloaded from the Protein Data Bank in PDB format accessible on the site (https://www.rcsb.org/). Proteins with PDB ID 3AYU for lung cancer [24] and PDB ID 2D60 for breast cancer [25], and other molecules and water, were cleaned using Biovia Discovery Studio software (Client Version 2021). Redocking studies were performed with the original protein and ligand prepared in a grid box measuring 52.24 × 38.88 × 42.59 Å (PDB ID 3AYU) and 43.79 × 36.97 × 37.85 Å (PDB ID 2D60) with a spacing of 0.375 Å for 100 runs of the generic Lamarck algorithm using AutoDock4.2 software. The redocking process was declared valid if the root mean square deviation (RMSD) value was < 2.00 Å. Molecular docking studies were performed using parameters similar to the redocking investigation [26]. Next, molecular docking was performed using AutoDock Vina software between the bioactive compound and the prepared protein. The molecular docking results were revisualized to observe the interactions and types of bonds that occurred using Biovia Discovery Studio [27, 28].

### 2.6. Statistical Analysis

Statistical analysis was performed on anticancer activity data using GraphPad Prism 10.0.1 software. The difference in the average significance of anticancer activity between test cells and types of isolates was performed using analysis of variance (ANOVA). Anticancer activity data are expressed as mean ± standard deviation (mean ± SD), with a significance level of *p* < 0.05, which is considered statistically significant (^∗^*p* < 0.05, ^∗∗^*p* < 0.01, ^∗∗∗^*p* < 0.001, and ^∗∗∗∗^*p* < 0.0001).

## 3. Results and Discussion

### 3.1. Preparation and Isolation of Bioactive Compounds From *C. amboinicus* Leaves

The sample was prepared with as much as 1.14 kg of dry material and extracted with ethanol solvent (pro analysis) using the maceration method for 2 days, and then remaceration was carried out with the same solvent and time. The extract was filtered and plated using a rotary vacuum evaporator at a temperature of 55°C to obtain a thick ethanol extract. The thick ethanol extract obtained was then partitioned with various types of solvent polarity sorted from *n*-hexane, chloroform, and ethyl acetate to obtain four types of extracts. Each extract obtained was then tested for anticancer activity using the MTT method against lung cancer cells (A549) and breast cancer cells (MCF-7) (Table 1).

The results of the activity test against lung cancer cells (A549) and breast cancer cells (MCF-7) from the initial ethanol extract obtained showed moderate anticancer activity and selectivity index (SI) exceeding 2, thus indicating good potential for use as a natural ingredient–based anticancer therapy. The *n*-hexane extract has moderate anticancer activity against breast cancer cells and an SI value > 2, and the EtOAc extract has moderate activity and an SI value > 2, which is used as the basis for determining the extract to proceed to the purification stage.

Purification of each extract was carried out by gravity column chromatography using silica gel 60 (0.0063–0.22 mm) (stationary phase) and a comparison eluent between *n*-hexane and ethyl acetate (mobile phase). The eluent began with the use of 100% *n*-hexane solvent, and the polarity gradually increased until the use of 100% EtOAc, with a total of 50 mL each. Each extract was purified by gravity column chromatography using column chromatography with a diameter of 1.5 cm and a silica height of 11 cm, and the eluate was collected in a glass vial bottle with a maximum volume of 10 mL. The collected eluates were then characterized and analyzed using various instruments such as FT-IR_(KBr)_, UV-Vis spectrometry, GC-MS, and ^1^H and ^13^C-NMR.

### 3.2. Characterization and Analysis of Active Isolates

The collected active isolates (eluates) were evaporated at room temperature to remove the eluent. Two eluates from the purification of *n*-hexane extract were obtained, namely, (1) in the form of a liquid paste (orange-brown in color; vial bottle no. 3) and (2) crystals (yellow-orange; vial bottle no. 30), while three eluates from the purification of EtOAc were obtained, namely, (1) in the form of a paste (orange in color; no. 5), and two isolates in the form of crystals (orange in color; vials 33 and 34). The eluates (active isolates) from each were continued to the melting point characterization stage with melting point (only isolates in the form of crystals), and determination of retention factor (Rf) values was done using TLC (Figure S1). Interpretation of the characterization data of the isolates obtained is described in Table 2.

Structure elucidation using FT-IR_(KBr)_ to analyze functional groups of each isolate is shown in Figure S2. Isolate a (I_nH-1_) shows absorption of functional groups C=O (1735.93 cm^−1^), -CH_2_- (1458.18 cm^−1^), -CH_3_ (2924.02 cm^−1^), and -CO- (1095.57–1172.72 cm^−1^); isolate b (I_nH-2_) shows absorption of functional groups -OH (3363.86 and 3479.58 cm^−1^), -CH_3_ (2924.02 cm^−1^), -CH_2_- (1442.75 cm^−1^), -CO- (1072.42–1165.00 cm^−1^), and -C=C- (1635.64 cm^−1^); isolate c (I_EtOAc-1_) showed absorption of functional groups -OH (3348.42 cm^−1^), -CH_3_ (2924.09 cm^−1^), -CO (carbonyl ketone, 1735.93 cm^−1^), and -CO- (methoxy, 1095-1172.72 cm^−1^); isolate d (I_EtOAc-2_) showed absorption of functional groups -OH (3348.42 cm^−1^ and 3479.58 cm^−1^), -CH_3_ (2924.09 cm^−1^), -CO (carbonyl ketone, 1705.07 cm^−1^), C=C (alkene, 1635.64 cm^−1^), and -CO- (methoxy, 1072.42 cm^−1^); isolate e (I_EtOAc-3_) showed absorption of functional groups -OH (3356.14 cm^−1^ and 3479.58 cm^−1^), -CH_3_ (2924.09 cm^−1^), -CO (carbonyl ketone, 1705.07 cm^−1^), C=C (alkene, 1635.64 cm^−1^), and -CO- (methoxy, 1072.42 cm^−1^). The results of the absorption of functional groups carried out on isolates (d) and (e) showed relatively the same absorption, so the assumption indicates that isolates (d) and (e) are the same.

### 3.3. Analysis of the Chemical Structure of the Isolate Using GC-MS

Analysis of the content of active compounds contained in each determination of molecular formula and levels using GC-MS is given in Figure 1 and Table 3. Active isolate (a) contains 40 types of compounds with the five highest levels of compounds, namely, squalene (13.23%); neophytadiene (10.51%); 5-eicosene (E) (10.1%); 1-octadecene (7.71%); and 1-tetracosene (7.09%). Isolate (b) contains 21 types of compounds with the five highest levels of compounds, namely, 2,3-pyrazinediamine, N3-cyclopropyl-N2, N2-dimethyl-5,6-diphenyl (72.84%); dehydroabietic acid (5.86%); 1,4,10,13-tetraazacyclooctadeca-4,9,13,18-tetraene-7,16-dione, 5,9,14,18-tetramethyl (4.48%); 4-[3-(4-amino-1,2,5-oxadiazol-3-yl)-6-fluoroquinoxalin-2-yl]-1,2,5-oxadiazol-3-amine (3.08%); and dibenz[b,d]cycloheptanone, 1,2,9-trimethoxy (2.91%). Isolate (c) contains 30 compounds, with the five highest compounds being hexadecanoic acid, ethyl ester (19.74%); 1,4-benzenedicarboxylic acid, bis(2-ethylhexyl) ester (14.63%); (Z,Z,Z) 9,12,15-octadecatrienoic acid, ethyl ester (12.31%); 2,4-Di-tert-butylphenol (9.67%); and 3,7,11,15-Tetramethylhexadec-2-en-1-yl acetate (9.46%). Isolate (d) contains 21 compounds, with the five highest compounds including Aflatoxin G2 (39.59%); 2-Pentadecanone, 6,10,14-trimethyl (14.81%); 1,2,3-Trimethoxy-5-[2-(4-methoxyphenyl)ethynyl]benzene (6.87%); 4-[3-(4-Amino-1,2,5-oxadiazol-3-yl)-6-fluoroquinoxalin-2-yl]-1,2,5-oxadiazol-3-amine (6.12%); and dibenz[d,f]cycloheptanone, 2,3,9-trimethoxy (5.95%). Isolate (e) contained 43 compounds with the five highest levels, namely, 2,3-pyrazinediamine, N3-cyclopropyl-N2, N2-dimethyl-5,6-diphenyl (14.54%); 2-pentadecanone, 6,10,14-trimethyl (13.07%); ethanone, 1-(2,2-dimethylcyclopentyl) (12.44%); valeric acid, 3-pentadecyl ester (5.24%); and sulfurous acid, hexyl undecyl ester (4.41%).

### 3.4. NMR-Based Chemical Structure Analysis of Active Isolates

Chemical structure analysis using ^1^H and ^13^C-NMR was carried out on all isolates to provide a comprehensive picture of the compound content of each isolate.  Figure S3 presents the ^1^H and ^13^C-NMR spectra of each active isolate from the purification of *n*-hexane and ethyl acetate extracts of *C. amboinicus* leaves. ^1^H and ^13^C-NMR analysis of isolate (a) shows the presence of organic structures with functional groups of methine, allylic, benzylic, alcohols, esters, carboxylic acids, alkenes, ethers, alcohols, esters, methyl, and methylene (Figure S3(a)). Isolate (b) shows the presence of organic structures with functional groups of methine, allylic, benzylic, alcohols, alkyl halides, esters, carboxylic acids, alkenes, ethers, alcohols, esters, methyl, and methylene, ketones, and aldehydes (Figure S3(b)). Isolate (c) showed the presence of functional groups methine, allylic, benzylic, alcohols, alkyl fluoride, esters, carboxylic acids, alkenes, ethers, alcohol, ester, methyl, methylene, aromatic, and ketone (Figure S3(c)). Isolated (d) showed the presence of functional groups methine, allylic, benzylic, alcohols, alkyl fluoride, esters, carboxylic acids, alkenes, ethers, alcohols, esters, methyl, methylene, aldehyde, and ketone (Figure S3(d)). Isolate (e) showed the presence of functional groups: methine, allylic, benzylic, alcohols, alkyl halides, esters, carboxylic acids, alkenes, ethers, alcohols, esters, methylene, methyl, and ketones (Figure S3(e)). These findings are in line with the results of the analysis using FT-IR_(KBr)_ and GC-MS of each active isolate. Each active isolate obtained was continued to in vitro cytotoxic testing on lung cancer cells (A549) and breast cancer cells (MCF-7).

### 3.5. Cytotoxic Test of Active Isolates Against A549 and MCF-7 Cells

**Figure 2:**
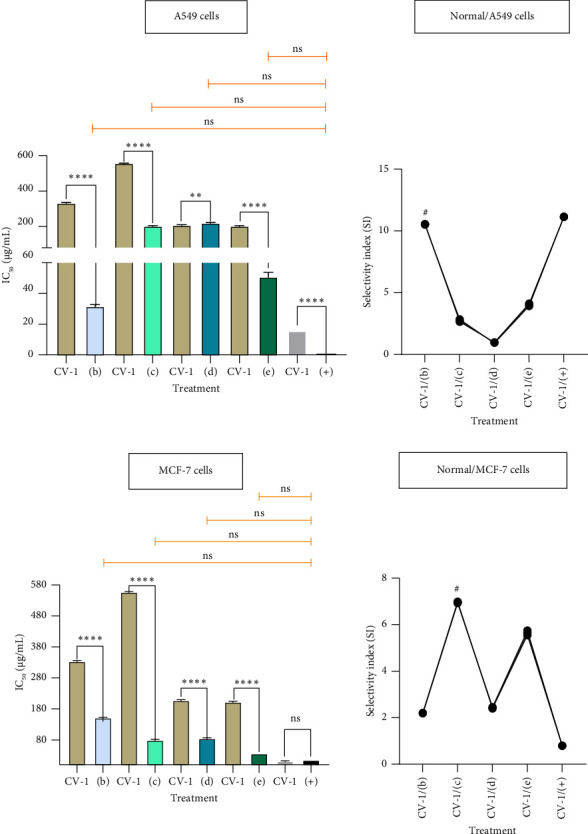
Data analysis of activity testing and selectivity index of each isolation against cancer cells; activity testing data are expressed as means ± SD with significance levels ^∗^*p* < 0.05, ^∗∗^*p* < 0.01, ^∗∗∗^*p* < 0.001, and ^∗∗∗∗^*p* < 0.0001. The ^#^ sign indicates the selected isolate to continue bioinformatics and molecular docking studies.

Active isolates (a) and (b) were purified from *n*-hexane extract, and isolates (c), (d), and (e) were purified from ethyl acetate extract. Each isolate was tested for in vitro cytotoxicity against lung cancer cells (A549), breast cancer cells (MCF-7), and normal cells (CV-1) using the MTT method. The National Cancer Institute (NCI) classifies the cytotoxic activity (IC_50_) values as follows: IC_50_ < 20 μg/mL in the high category, IC_50_ 20–100 μg/mL in the moderate category, IC_50_ 101–500 μg/mL in the weak category, and IC_50_ > 500 μg/mL in the inactive category as an anticancer [29, 30]. Isolate (a) did not show cytotoxic activity against A549 and MCF-7 cancer cells and normal cells (IC_50_ > 1000 μg/mL); isolate (b) showed IC_50_ of 31.74 ± 1.02 μg/mL against A549 cells (moderate category), 152.80 ± 1.00 μg/mL against MCF-7 cells (weak category), and 334.20 ± 1.99 μg/mL against normal cells (weak category); isolate (c) showed IC_50_ of 203.3 ± 1.98 μg/mL against A549 cells (weak category), 80.05 ± 1.99 μg/mL against MCF-7 cancer cells (moderate category), and 557.50 ± 1.02 μg/mL against normal cells (inactive category); isolate (d) showed IC_50_ of 221.30 ± 2.03 μg/mL against A549 cells (weak category), 85.32 ± 1.96 μg/mL against MCF-7 cells (moderate category), and 207.50 ± 1.97 μg/mL against normal cells; and isolate (e) showed IC_50_ of 50.93 ± 1.75 μg/mL against A549 cells (moderate category), 36.26 ± 0.01 μg/mL against MCF-7 cells (moderate category), and 204.90 ± 1.92 μg/mL against normal cells (weak category) (Figure 2). The positive control used in the cytotoxic test against A549 cells was doxorubicin, and the positive control in the test against MCF-7 cells was cisplatin. The IC_50_ value of doxorubicin against A549 cells is 1.42 ± 0.01 μg/mL and 15.82 ± 0.01 μg/mL against CV-1 cells, both showing strong category activity. The IC_50_ value of cisplatin against MCF-7 cells is 12.6 ± 0.10 μg/mL and 15.90 ± 0.01 μg/mL against CV-1 cells, both showing strong category activity. The results of testing the drug on both cancer cells (A549 cells and MCF-7 cells) and normal cells (CV-1) both showed strong category cytotoxic activity. This is what causes the bad side effects caused by drugs on normal/healthy cells in cancer treatment. Previous studies have also revealed severe side effects from the use of doxorubicin drugs, including cardiotoxicity, hepatotoxicity, nephrotoxicity, and gonadotoxicity [31, 32]. Meanwhile, the side effects caused using the cancer drug cisplatin are cytopenia, hematological toxicity, anaphylaxis, hepatotoxicity, cardiotoxicity, ototoxicity, nausea and vomiting, mucositis, diarrhea, stomatitis, alopecia, anorexia, cachexia, pain, and asthenia [33, 34].

**Figure 3:**
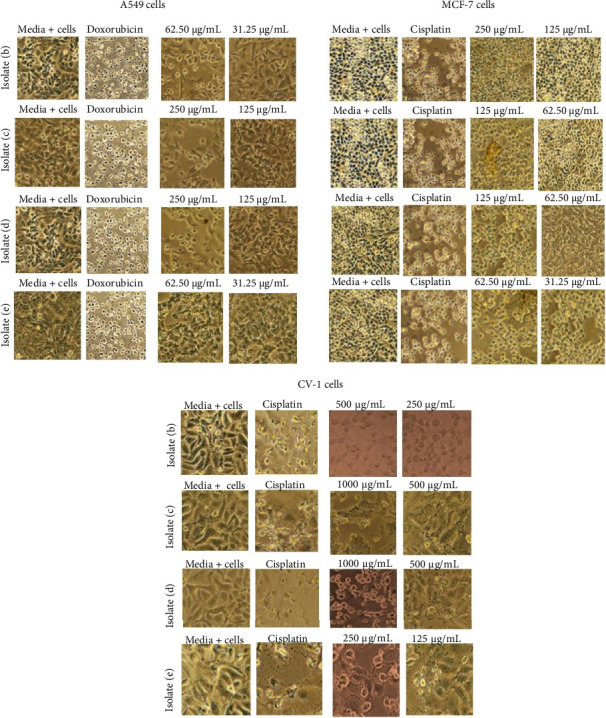
Morphology of activity testing of each isolate against cancer cells (A549 and MCF-7) and normal cells (CV-1).

Important information that is a finding based on further statistical analysis of the IC_50_ value of compound (b) shows a very significant (*p* < 0.0001) difference between A549 cells and CV-1 cells. This information shows that compound (b) is more effective against A549 cells than CV-1 cells, but its activity has not been able to replace the drug (doxorubicin) as a positive control. The same thing applies to compound (c), where compound (c) is more effective against MCF-7 cells than CV-1 cells (*p* < 0.0001), and its activity has not been able to replace the drug (cisplatin) as a positive control. Recommended active compounds can be used as cancer drugs, namely, they must have good activity values against cancer, be selective, and must also be inactive or have weak activity against normal cells (low side effects). The basis for consideration of cytotoxicity in cancer cells and normal cells can be determined from the SI value. Active compounds that have the potential to be developed as cancer drugs must have an SI value > 3 [35, 36]. Compound (b) has potential activity against A549 cancer cells, and compound (c) has potential against MCF-7 cancer cells. The results of these findings provide an alternative recommendation to be used as an active compound for anticancer agents in the development of related cancer drugs. The morphology of the results of the anticancer activity test of each isolate against cancer cells at the concentration interval of the IC_50_ value area is shown in Figure 3.

### 3.6. Bioinformatics Study With a Network Pharmacology Approach

**Figure 4:**
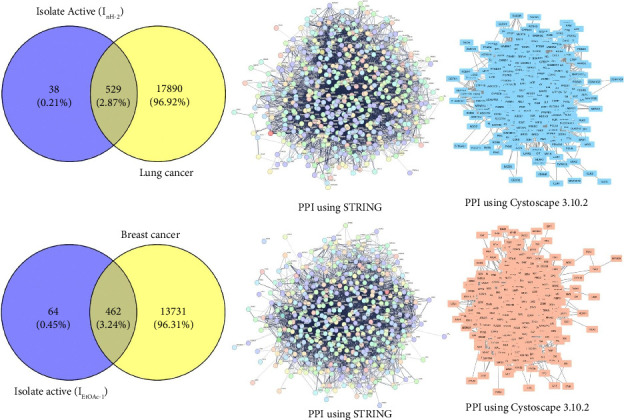
Analysis of genes of each bioactive compound isolate and target disease gene and their interactions.

The selected active isolates were continued with bioinformatic study with a network pharmacology approach. Active isolate (I_nH-2_) was selected for lung cancer with a total of 22 compounds, and active isolate (I_EtOAc-1_) for breast cancer with 30 compounds. The active compounds from each isolate used in the bioinformatic study were the results of isolate identification and analysis using GC-MS. The use of all compounds in the bioinformatic study with the network pharmacology approach aims to obtain comprehensive information on the main proteins involved in the use of targeted cancer therapy. The collection of bioactive compound genes for each isolate used the SwissPredictionTarget database, and the target disease genes were obtained from GeneCards according to the procedure described in the previous section. The total genes of all compounds from the screening results for active isolate (I_nH-2_) were 567 genes and 18419 genes from lung cancer, while the screening genes for active isolate (I_EtOAc-1_) were 526 genes and 14193 genes from breast cancer. Each active isolate gene was integrated with the target cancer with a Venn diagram to obtain overlapping genes between the active isolate and the target cancer as potential genes and then continued with PPI analysis (Figure 4).

**Figure 5:**
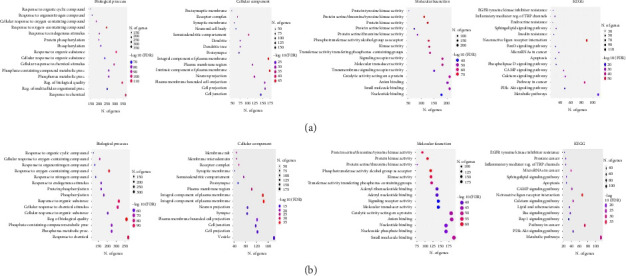
Gene Ontology and KEGG enrichment analysis: (a) active isolate (I_nH-2_) related to lung cancer and (b) active isolate (I_EtOAc-1_) related to breast cancer.

Analysis of the biological function of the potential target pathway of each active isolate against the target cancer was continued by conducting GO functional enrichment analysis, including BPs, CCs, and MFs, as well as KEGG pathway enrichment analysis using an FDR cutoff of 0.05 from each active isolate that is closely related to the target cancer. Each of the three GO functional enrichments of the active isolate (I_nH-2_) is related to lung cancer; BP enrichment includes response to organic cyclic compound, response to organonitrogen compound, and cellular response to oxygen-containing compound; CC includes postsynaptic membrane, synaptic membrane, and receptor complex; MF includes protein tyrosine kinase activity, protein serine/threonine/tyrosine kinase activity, and protein kinase activity. KEGG pathway enrichment analysis of 529 overlapping target genes resulted in 242 related pathways, especially cancer pathway studies. GO functional enrichment of active isolate (I_EtOAc-1_) related to breast cancer; BP enrichment includes response to organic cyclic compound, cellular response to oxygen-containing compound, and response to organonitrogen compound; CC includes membrane raft, membrane microdomain, and receptor complex; MF includes protein serine/threonine/tyrosine kinase activity, protein kinase activity, and protein serine/threonine kinase activity, and KEGG pathway enrichment analysis of 462 overlapping target genes resulted in 245 related pathways, especially cancer pathway studies (Figure 5).

**Figure 6:**
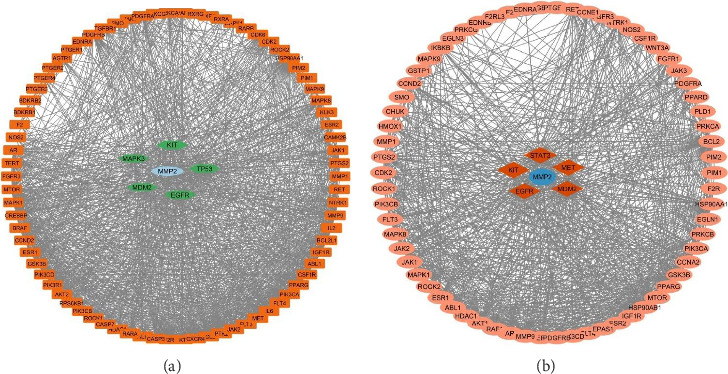
Genes involved in KEGG enrichment of cancer pathways: (a) active isolate (I_nH-2_) related to lung cancer and (b) active isolate (I_EtOAc-1_) related to breast cancer.

Further molecular studies of each isolate against the target cancer are focused on the molecular cancer pathway. Genes involved in the cancer pathway are filtered based on average shortest path length, betweenness centrality, closeness centrality, clustering coefficient, eccentricity, edge count, and indegree to obtain one target gene that is the main protein in the validation of molecular-protein interactions with molecular docking. Genes from each active isolate involved in the cancer pathway are given in Figure 6 and Table 4. Genes involved in the KEGG molecular pathway in cancer on the target lung cancer are 84 genes, and in breast cancer, 72 genes. The main protein in the validation of molecular-protein interactions from active isolate (I_nH-2_) against lung cancer is the matrix metalloproteinase-2 (MMP-2) protein, and the active isolate (I_EtOAc-1_) against breast cancer is the MMP-2 protein. Active isolates (I_nH-2_) and (I_EtOAc-1_) have the same target protein, namely, MMP-2 from KEGG enrichment in the cancer pathway. MMP-2 functions to regulate the migration and invasion of lung cancer cells into blood, cell proliferation, and metastasis of cancer cells in general vessels [37, 38], as well as being an excellent biomarker for early diagnosis of lung cancer, differentiating cancer types, and determining disease stages [39]. This protein, also in breast cancer types, has a protective role in cancer development as well as an opposing role depending on the type of cell where the MMP is expressed or the stage of cancer [40].

### 3.7. Molecular Docking Studies

The bioactive compounds used in validation by molecular docking against the main protein of each isolate were five compounds with the highest levels of GC-MS analysis results. The five compounds were predicted by ADMET using the database (https://www.swissadme.ch/ and https://tox.charite.de/protox3/index.php?site=home), energy optimization of the compound structure using GaussView 5.0.8 software with DFT method settings, and basis set 3-21G (Table 5).

The results of the energy optimization of active compounds show that all active compounds have negative energy, indicating that the compound is stable. The lower the value of the compound optimization energy, the better the stability. The dipole moment indicates the polarity of the bond between atoms in the molecule, where the greater the value of the dipole moment, the higher the polarity of the bond. The content of the active isolate compound (I_nH-2_), which has a more stable bond, is compound 4 (4-[3-(4-amino-1,2,5-oxadiazol-3-yl)-6-fluoroquinoxalin-2-yl]-1,2,5-oxadiazol-3-amine (content 3.08%) with a dipole moment of 9.62. The content of the active isolate (I_EtOAc-1_), which has a more stable bond in the compound, is the compound 1,4-benzenedicarboxylic acid, bis(2-ethylhexyl) ester (content 14.63%) with a dipole moment of 0.50. Based on the ADMET drug discovery rules, there are five established Lipinski's rules, including (i) molecular mass less than 500 Daltons, (ii) high lipophilicity (Log*p* < 5), (iii) hydrogen bond donor < 5, (iv) hydrogen bond acceptor < 10, and (v) molar refractivity between 40 and 130 [41, 42]. Then all active isolate compounds (I_nH-2_) meet, and compound 4 of the active isolate (I_EtOAc-1_) does not meet the Lipinski rule due to limitations of the database used (data not available). Lipinski's rule of five is used as a guide for oral drug use, and if it exceeds the limit or does not comply with the existing rules, it is estimated that the activity of drugs consumed orally will be poor. Compliance with the rules in oral drug development creates effective success, optimal potential activity, good specificity, and selectivity [43].

Molecular docking of active compounds from each isolate was carried out against the same protein, namely, MMP-2, with PDB ID 3AYU for lung cancer, which reveals the effectiveness of proliferation by reducing PCNA, inducing lung cancer apoptosis through activation of the caspase-dependent apoptosis pathway, and inhibiting lung cancer migration [24]. PDB ID 2D60 for breast cancer reveals the effectiveness of antiproliferative inhibition in breast cancer [25]. The redocking process was performed to evaluate the validity of the docking protocol with the native ligand in the active site of the receptor. During this process, the conformation of the native ligand did not differ significantly from the initial conformation because the initial conformation has the most stable complex structure in line with the experimental data. The superimposed structure of the native ligand in the active site of the receptor is shown in Figure S4. Figure S4 shows that there is no significant change between the initial and final conformations after the redocking process. The RMSD values of the redocking process were found to be 0.76 Å (PDB ID 3AYU) and 0.75 Å (PDB ID 2D60). These RMSD values are acceptable because they are less than 2.00 Å, as required in the redocking process [26].

**Figure 7:**
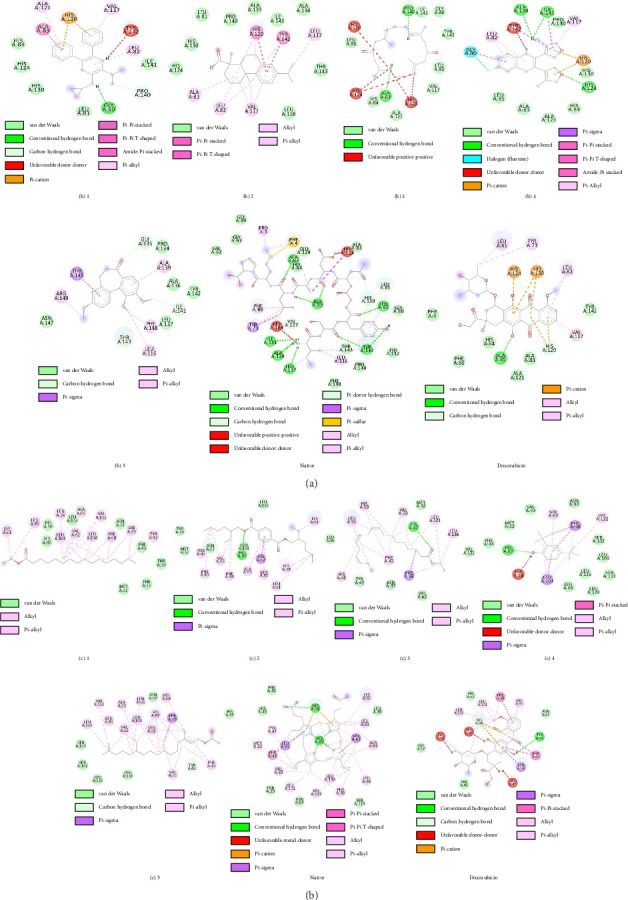
Interaction of active isolate compounds with MMP-2 protein: (a) lung cancer and (b) breast cancer.

Molecular docking studies of each active compound in the active isolate with the main protein of the target cancer are presented in Table 6 and Figures 7(a) and 7(b). The content of the isolated active compound (InH-2) with the active compound code (b)2 shows a more stable binding stability (binding energy −9.5 kcal/mol, binding constant 0.17 μM, RMSD 0.03 Å) compared to the original ligand (binding energy −8.2 kcal/mol, binding constant 0.17 μM, RMSD 0.76 Å) and the cancer drug doxorubicin (binding energy −7.7 kcal/mol, binding constant 0.17 μM, RMSD 0.07 Å), so it is likely to show better interaction against inhibition of proliferation, induction of apoptosis, and migration of lung cancer cells. The active isolate (IEtOAc-1) with compound code (c)1 (binding energy −8.3 kcal/mol, binding constant 0.39 μM, RMSD 1.30 Å) and (c)2 (binding energy −9.4 kcal/mol, binding constant 0.12 μM, RMSD 0.96 Å) has better binding stability compared to the original ligand (binding energy −7.8 kcal/mol, binding constant 0.09 μM, RMSD 1.00 Å) and doxorubicin drug (binding energy −7.8 kcal/mol, binding constant 0.58 μM, RMSD 0.54 Å), so this compound is also suspected to have better ability in inhibiting proliferation in breast cancer therapy.

## 4. Conclusion

The active compounds from each isolate of bangun-bangun (*Coleus amboinicus*, Lour.) leaves were successfully identified from each extract of the initial maceration ethanol extract partition results. The *n*-hexane and ethyl acetate extracts showed the best activity against lung cancer and breast cancer based on the MTT method. The extracts were the basis for purification to obtain active isolates. The active isolate (InH-2) from the *n*-hexane extract showed the best activity against lung cancer cells/A549, and the active isolate (IEtOAc-1) from the ethyl acetate extract showed the best activity against breast cancer cells/MCF-7 and showed no toxicity to normal cells (CV-1). Network pharmacology studies on potential isolated pathways in cancer target MMP-2 proteins in lung and breast cancer therapy. Molecular docking studies show that the active compound from isolate (InH-2) with code (b)2 has a more stable binding compared to the original ligand and doxorubicin (cancer drug), while the active isolate (IEtOAc-1) with compound code (c)1 has a more stable binding compared to the original ligand and doxorubicin. These compounds are thought to have better ability in inhibiting proliferation in lung and breast cancer therapy. Further research and development are still needed, especially in obtaining active compounds in single and pure form and further testing to obtain potential active isolates in the treatment or therapy of lung and breast cancer.

## Figures and Tables

**Table 1:** Results of the extraction and partition process of *C. amboinicus* leaf samples.

No.	Treatment		Initial EtOH crude extract (g)	Extract partition (g)			
				*n*-C_6_H_12_^a^	CHCl_3_	EtOAc^a^	Residual (EtOH-H_2_O)
1	Dry sample (1.14 kg)		108.01 ± 0.01^∗^	36.21 ± 0.01	18.01 ± 0.02	14.11 ± 0.02	11.61 ± 0.03

2	% Yield		9.48 ± 0.01^∗∗^	45.27 ± 0.03^∗∗∗^	22.53 ± 0.01^∗∗∗^	17.66 ± 0.01^∗∗∗^	14.53 ± 0.01^∗∗∗^

3	IC_50_ (μg/mL)	A549 cell	212.80 ± 0.10	544.80 ± 0.15	251.20 ± 0.20	554.60 ± 0.30	554.10 ± 0.15
		MCF-7 cell	154.80 ± 0.21	217.20 ± 0.17	102.30 ± 0.26	108.00 ± 0.10	396.40 ± 0.32

4	Selectivity index (SI)	CV-1/A549	2.36 ± 0.01	1.16 ± 0.01	0.75 ± 0.02	0.82 ± 0.02	0.96 ± 0.01
		CV-1/MCF-7	3.24 ± 0.01	2.91 ± 0.02	1.85 ± 0.01	4.23 ± 0.02	1.34 ± 0.03

*Note:* Data are presented as means ± SD with *n* = 3.

^∗^The weight of the partitioned extract was 80.01 g.

^∗∗^Weight of extract compared with weight of dry sample.

^∗∗∗^The partitioned extract was compared with the initial extract weight that was continued (80.01 g).

^a^Purified extract.

**Table 2:** Characterization of active isolates from column chromatography of *n*-hexane extract and EtOAc extract of *C. amboinicus* leaves.

Types of extracts	Active isolates	Vial position	Solubility	Melting point	TLC				Electronic transition analysis using UV-Vis spectrophotometry (nm)
					Eluent *n-*C_6_H_12_:EtOAc		Observation		
					7:3	9:1	254 nm	UV	
*n*-C_6_H_12_	I_nH_-1	3	*n*- C_6_H_12_, EtOAc	—	0.78	0.76	Black	Brown	217; 227; 243; 248; 253; 258; 261; 263; 265; 272; 277; 280; 282; 288; 294; 323
	I_nH_-2	30	*n*- C_6_H_12_, EtOAc, EtOH	164–166	0.88	0.38	Black	Brown	274; 403

EtOAc	I_EtOAc_-1	5	*n*- C_6_H_12_, EtOAc	—	0.96	0.86	Black	Brown	195; 205; 252; 254; 259; 262; 267; 273; 275; 277; 282; 324
	I_EtOAc_-2	33	*n*- C_6_H_12_, EtOAc, EtOH	162–164	0.94	0.44	Black	Brown	275; 404
	I_EtOAc_-3	34			0.94	0.46	Black	Brown	275; 404

**Table 3:** Analysis of each component of chemical compounds from active isolates using GC-MS.

**No.**	**Retention time (RT)**	**% area**	**Compound name (a)**	**Molecular formula**	***m*/*z* (g/mol)**

1	13.3	0.25	Copaene	C_15_H_24_	204.35
2	13.4	0.66	1-Tetradecene	C_14_H_28_	196.37
3	14.7	0.33	4a,8-Dimethyl-2-(prop-1-en-2-yl)-1,2,3,4,4a,5,6,7-octahydronaphthalene	C_15_H_24_	204.35
4	14.7	0.29	Pentadecane	C_15_H_32_	212.41
5	15.1	0.64	Naphthalene, 1,2,3,5,6,8a-hexahydro-4,7-dimethyl-1-(1-methylethyl)-, (1S-cis)	C_15_H_24_	204.35
6	15.1	0.31	Naphthalene, 1,2,3,4-tetrahydro-1,6-dimethyl-4-(1-methylethyl)-, (1S-cis)	C_15_H_22_	202.34
7	15.9	4.15	Cetene	C_15_H_22_	224.42
8	15.9	1.12	Hexadecane	C_16_H_34_	226.44
9	17	0.22	7-Hexadecene, (Z)-	C_16_H_32_	224.4253
10	17	2.33	Naphthalene, 1,6-dimethyl-4-(1-methylethyl)	C_15_H_18_	198.3
11	17.1	1.27	Oxalic acid, cyclohexylmethyl tridecyl ester	C_22_H_40_O_4_	368.5
12	18.1	7.71	5-Octadecene, (E)-	C_18_H_36_	252.5
13	18.3	1.09	1-Octadecene	C_18_H_36_	252.48
14	18.6	10.51	Tetratetracontane	C_44_H_90_	619.19
15	19.2	4.31	Neophytadiene	C_20_H_38_	278.5
16	19.4	3.98	Nonadecane	C_19_H_40_	268.5
17	19.6	0.72	Pentadecafluorooctanoic acid, octadecyl ester	C_26_H_37_F_15_O_2_	666.5
18	19.8	1.84	Isopimara-9(11),15-diene	C_20_H_32_	272.5
19	20	0.59	Isolongifolene, 9,10-dehydro-	C_15_H_22_	202.33
20	20.1	0.38	2-Hexadecene, 2,6,10,14-tetramethyl	C_20_H_40_	280.5
21	20.2	10.1	Hexanoic acid, 4-hexadecyl ester	C_22_H_44_O_2_	340.6
22	20.2	4.23	5-Eicosene, (E)-	C_20_H_40_	280.5
23	20.5	0.31	Eicosane	C_20_H_42_	282.5
24	20.5	0.31	Umbelliprenin	C_24_H_30_O_3_	366.5
25	20.6	0.46	4b,8-Dimethyl-2-isopropylphenanthrene, 4b,5,6,7,8,8a,9,10-octahydro	C_19_H_28_	256.3
26	21	1.52	Phenanthrene, 1,2,3,4,4a,9,10,10a-octahydro-1,1,4a-trimethyl-7-(1-methylethyl)-, (4aS-trans)	C_20_H_30_	270.5
27	21.2	0.4	Heneicosane	C_21_H_44_	296.6
28	21.4	0.35	Methyl stearate	C_19_H_38_O_2_	298.5
29	21.6	0.36	3-Octadecene, (E)-	C_18_H_36_	252.5
30	22	7.09	1-Tetracosene	C_24_H_48_	336.6
31	22.3	0.25	1-Docosene	C_22_H_44_	308.6
32	23	1.2	Heptadecane	C_17_H_36_	240.5
33	23.7	4.0	Cyclotetracosane	C_24_H_48_	336.6
34	24.6	2.63	Pentacosane	C_25_H_52_	352.7
35	25.3	2.03	1-Hexacosene	C_26_H_52_	364.7
36	26.84	1.15	Octacosane	C_28_H_58_	394.8
37	27	13.23	Squalene	C_30_H_50_	410.7
38	27.6	6.74	Heptadecane, 9-octyl-	C_25_H_52_	352.7
39	28.4	0.56	Triacontane	C_30_H_62_	422.8
40	29.3	2.39	Hentriacontane	C_31_H_64_	436.8

**No.**	**Retention time (RT)**	**% area**	**Compound name (b)**	**Molecular formula**	***m*/*z* (g/mol)**

1	15.884	0.50	Diethyl phthalate	C_12_H_14_O_4_	222.24
2	16.778	0.28	α -cadinol	C_15_H_26_O	222.37
3	18.681	0.49	2-Pentadecanone, 6,10,14-trimethyl	C_18_H_36_O	268.5
4	19.854	1.77	*n*-Hexadecanoic acid	C_16_H_32_O_2_	256.42
5	21.013	0.22	2-Phenanthrenol, 1,2,3,4,4a,9,10,10a-octahydro-7-methoxy-1,1,4a-trimethyl	C_18_H_26_O_2_	274.4
6	21.316	0.24	Phytol	C_20_H_40_O	296.5
7	21.505	0.16	Linoelaidic acid	C_18_H_32_O_2_	280.4
8	21.568	0.64	9,12,15-Octadecatrienoic acid, (Z,Z,Z)	C_18_H_30_O_2_	278.4
9	21.605	0.51	Cyclopentane, 1,2,3,4,5-pentamethyl	C_10_H_20_	140.27
10	22.500	0.38	N1-(4-Imidazo[1,2-a] pyridin-2-ylphenyl) acetamide	C_15_H_13_N_3_O	251.28
11	23.042	0.41	4-Bromothiophenol, S-(2-methylpropionyl)		
12	23.269	0.28	1-Phenanthrenemethanol, 1,2,3,4,4a,9,10,10a-octahydro-1,4a-dimethyl-7-(1-methylethyl)-, [1r-(1α.,4adimethyl-7-(1-methylethyl)-, (1R-(1α., 4aβ., 10a. α))	C_20_H_30_O	286.5
13	23.445	1.33	4,8,12,16-Tetramethylheptadecan-4-olide	C_21_H_40_O_2_	324.5
16	23.660	3.08	4-[3-(4-Amino-1,2,5-oxadiazol-3-yl)-6-fluoroquinoxalin-2-yl]-1,2,5-oxadiazol-3-amine	C_12_H_7_FN_8_O_2_	314.24
14	23.761	1.05	3-(4-Chlorophenyl)-9-fluoro-[1,2,4] triazolo[4,3-c]quinazoline	C_15_H_8_ClFN_4_	298.7
15	23.836	2.91	Dibenz[b,d]cycloheptanone, 1,2,9-trimethoxy	C_18_H_18_O_4_	298.3
17	24.063	5.86	Dehydroabietic acid	C_20_H_28_O_2_	300.4
18	24.340	1.02	2-(3-Hydroxy-4-methoxyphenyl)-3-methoxy-4H-chromen-4-one	C_17_H_14_O_5_	298.29
19	25.298	72.84	2,3-Pyrazinediamine, N3-cyclopropyl-N2, N2-dimethyl-5,6-diphenyl	C_21_H_22_N_4_	330.4
20	25.462	4.84	1,4,10,13-Tetraazacyclooctadeca-4,9,13,18-tetraene-7,16-dione, 5,9,14,18-tetramethyl	C_18_H_28_N_4_O_2_	332.4
21	27.252	0.22	α-Tocospiro A	C_29_H_50_O_4_	462.7

**No.**	**Retention time (RT)**	**% area**	**Compound name (c)**	**Molecular formula**	***m*/*z* (g/mol)**

1	14.838	9.67	2,4-Di-tert-butylphenol	C_15_H_24_	204.35
2	15.859	0.30	Cetene	C_16_H_32_	224.4
3	17.106	0.49	5-(2-Thienyl) pentanoic acid	C_9_H_12_O_2_S	184.2
4	18.114	0.66	Tetradecanoic acid, ethyl ester	C_15_H_24_	204.35
5	18.581	6.4	Neophytadiene	C_20_H_38_	278.5
6	18.631	1.47	2-Pentadecanone, 6,10,14-trimethyl-	C_18_H_36_O	268.5
7	19.16	0.32	Ethyl 13-methyl-tetradecanoate	C_17_H_34_O_2_	270.5
8	19.375	0.27	8-Nonen-2-one	C_9_H_16_O	140.22
9	19.488	1.73	Hexadecanoic acid, methyl ester	C_17_H_34_O_2_	270.5
10	19.564	0.25	Benzenepropanoic acid, 3,5-bis(1,1-dimethylethyl)-4-hydroxy-, methyl ester	C_18_H_28_O_3_	292.4
11	19.791	19.74	Hexadecanoic acid, ethyl ester	C_18_H_36_O_2_	284.5
12	19.954	0.26	Ethyl 9-hexadecenoate	C_18_H_34_O_2_	282.5
13	20.081	1.65	E-11-Hexadecenoic acid, ethyl ester	C_18_H_34_O_2_	282.5
14	20.849	0.95	Heptadecanoic acid, ethyl ester	C_19_H_38_O_2_	298.5
15	21.19	0.59	11-Octadecenoic acid, methyl ester	C_19_H_36_O_2_	296.5
16	21.278	0.49	Phytol	C_20_H_40_O	296.5
17	21.416	0.40	Methyl stearate	C_19_H_38_O_2_	298.5
18	21.568	0.34	4,4,6-Trimethyl-cyclohex-2-en-1-ol	C_9_H_16_O	140.22
19	21.744	6.70	Linoleic acid ethyl ester	C_20_H_36_O_2_	308.5
20	21.807	12.31	9,12,15-Octadecatrienoic acid, ethyl ester (Z, Z, Z)	C_20_H_34_O_2_	306.5
21	22.021	5.85	Octadecanoic acid, ethyl ester	C_20_H_40_O_2_	312.5
22	22.198	9.46	3,7,11,15-Tetramethylhexadec-2-en-1-yl acetate	C_22_H_42_O_2_	338.6
23	22.891	0.16	Nonanoic acid, 2,4,6-trimethyl-,methyl ester, (2R,4S,6R)-(−)	C_13_H_26_O_2_	214.34
24	23.597	0.47	4-[3-(4-Amino-1,2,5-oxadiazol-3-yl)-6-fluoroquinoxalin-2-yl]-1,2,5-oxadiazol-3-amine	C_12_H_7_FN_8_O_2_	314.24
25	23.748	1.76	Eicosanoic acid, ethyl ester	C_22_H_44_O_2_	340.6
26	24.29	0.86	2,4-Diamino-6-[[m-methoxyphenyl] thio] quinazoline	C_15_H_14_N_4_OS	298.4
27	24.58	0.25	4,6-Bis (Morpholin-4-yl)-2,1,3-benzoxadiazole	C_14_H_18_N_4_O_3_	290.32
28	25.336	0.30	Docosanoic acid, ethyl ester	C_24_H_48_O_2_	368.6
29	26.458	14.63	1,4-Benzenedicarboxylic acid, bis(2-ethylhexyl) ester	C_24_H_38_O_4_	390.6
30	26.949	1.28	Squalene	C_30_H_50_	410.7

**No.**	**Retention time (RT)**	**% area**	**Compound name (d)**	**Molecular formula**	***m*/*z* (g/mol)**

1	14.245	0.29	2,6,10-Trimethyltridecane	C_16_H_34_	226.44
2	16.917	0.48	Cyclohexanol, 3-(aminomethyl)-3,5,5-trimethyl	C_10_H_21_NO	171.28
3	18.644	14.81	2-Pentadecanone, 6,10,14-trimethyl	C_18_H_36_O	268.5
4	19.11	0.77	Tetradecane	C_14_H_30_	198.39
5	19.39	1.72	Carbonic acid, hexadecyl prop-1-en-2-yl ester	C_20_H_38_O_3_	326.5
6	19.45	3.74	Ethanone, 1-(2,2-dimethylcyclopentyl)	C_9_H_16_O	140.22
7	20.18	1.32	3-Eicosene, (E)-	C_20_H_40_	280.5
8	21.01	0.50	1,3,2-Oxazaborolane, 2-butyl-	C_6_BNO	127.0
9	21.19	0.59	Malonic acid, 3,3-dimethylbut-2-yl heptadecyl ester	C_26_H_50_O_4_	426.7
10	21.57	0.41	1H-Imidazole, 2-heptadecyl-4,5-dihydro-, monoacetate	C_22_H_44_ON_2_O_2_	368.6
11	21.88	0.39	5,16-Androstadien-3beta-ol	C_19_H_28_O	272.0
12	22.05	0.53	Heptafluorobutyric acid, hexadecyl ester	C_20_H_33_F_7_O_2_	438.5
13	23.61	6.12	4-[3-(4-Amino-1,2,5-oxadiazol-3-yl)-6-fluoroquinoxalin-2-yl]-1,2,5-oxadiazol-3-amine	C_12_H_7_FN_8_O_2_	314.24
14	23.76	5.23	5-Isopentyl-6-methyl-2-(methylsulfanyl)-4-pyrimidinol,	C_11_H_18_N_2_OS	226.34
15	23.85	4.97	2,4-Diamino-5-[3-trifluoromethyl-4-methoxybenzyl] pyrimidine	C_13_H_13_F_3_N_4_O	298.26
16	24.0	5.95	Dibenz[d,f]cycloheptanone, 2,3,9-trimethoxy	C_18_H_18_O_4_	298.3
17	24.29	6.87	1,2,3-Trimethoxy-5-[2-(4-methoxyphenyl) ethynyl] benzene	C_18_H_18_O_4_	298.3
18	24.38	1.40	13-Methyl-tetradec-13-ene-1,12-diol	C_15_H_30_O_2_	242.40
19	24.59	0.63	Tridecane, 7-methyl	C_14_H_30_	198.39
20	25.21	39.59	Aflatoxin G2	C_17_H_14_O_7_	330.29
21	25.39	3.69	1,4,10,13-Tetraazacyclooctadeca-4,9,13,18-tetraene-7,16-dione, 5,9,14,18-tetramethyl	C_18_H_28_N_4_O_2_	332.4

**No.**	**Retention time (RT)**	**% area**	**Compound name (e)**	**Molecular formula**	***m*/*z* (g/mol)**

1	4.364	0.76	Hexanal	C_6_H_12_O	100.16
2	7.969	0.46	1-Hexanol, 2-ethyl	C_8_H_18_O	130.22
3	11.586	0.30	2-Decenal, (E)-	C_10_H_18_O	154.25
4	14.220	0.96	2,6,10-Trimethyltridecane	C_16_H_34_	226.44
5	15.846	1.67	Diethyl phthalate	C_12_H_14_O_4_	222.24
6	16.413	0.45	9-Eicosene, (E)	C_20_H_40_	280.5
7	16.791	0.85	Oxalic acid, isohexyl pentyl ester	C_13_H_24_O_4_	244.33
8	16.904	0.38	(1aR,3aS,7S,7aS,7bR)-1,1,3a,7-Tetramethyldecahydro-1H-cyclopropa[a]naphthalen-7-ol	C_15_H_26_O	222.37
9	17.106	0.97	2H-Tetrazole, 5-(thiophen-2-yl) methyl-	C_6_H_6_N_4_S	166.21
10	17.396	0.32	Cycloundecane, 1,1,2-trimethyl-	C_14_H_28_	196.37
11	18.114	2.02	1-Nonadecene	C_19_H_38_	266.5
12	18.581	0.27	Neophytadiene	C_20_H_38_	278.5
13	18.644	13.07	2-Pentadecanone, 6,10,14-trimethyl	C_18_H_36_O	268.5
14	19.110	0.92	(S)-5-Hydroxymethyl-2[5H]-furanone	C_5_H_6_O_3_	114.1
15	19.387	1.55	Carbonic acid, octadecyl prop-1-en-2-yl ester	C_22_H_42_O_3_	354.6
16	19.450	12.44	Ethanone, 1-(2,2-dimethylcyclopentyl)	C_9_H_16_O	140.22
17	19.841	2.32	n-Hexadecanoic acid	C_16_H_32_O_2_	256.42
18	19.942	1.00	Heptadecane, 9-octyl	C_25_H_52_	352.7
19	20.169	2.97	5-Eicosene, (E)-	C_20_H_40_	280.5
20	20.433	1.41	Octacosyl acetate	C_30_H_60_O_2_	452.8
21	20.950	0.82	Sulfurous acid, octadecyl pentyl ester	C_23_H_48_O_3_S	404.7
22	21.013	4.41	Sulfurous acid, hexyl undecyl ester	C_17_H_36_O_3_S	320.5
23	21.202	5.24	Valeric acid, 3-pentadecyl ester	C_20_H_40_O_2_	312.5
24	21.240	1.64	Ethyl 7-(2-oxocyclopentyl) heptanoate	C_14_H_24_O_3_	240.34
25	21.568	2.75	1-Benzoxepin-2(3H)-one, octahydro-; Octahydro-1-benzoxepin-2(3H)-one	C_10_H_16_O_2_	168.23
26	21.832	0.37	4-Methyl-E-9-octadecene	C_19_H_38_	266.5
27	22.185	2.04	Dimethyl(octyl)silyloxycyclohexane	C_16_H_34_OSi	270.53
28	22.299	0.46	Cedrol	C_15_H_26_O	222.37
29	22.614	0.79	Triacontan-15-ol	C_30_H_62_O	438.8
30	23.168	0.45	Tetratriacontyl pentafluoropropionate	C_37_H_69_F_5_O_2_	640.9
31	23.269	0.43	Dotriacontyl pentafluoropropionate	C_35_H_65_F_5_O_2_	612.9
32	23.534	0.33	9(11)-Dehydrotestosterone	C_19_H_26_O_2_	286.4
33	23.622	4.07	4-[3-(4-Amino-1,2,5-oxadiazol-3-yl)-6-fluoroquinoxalin-2-yl]-1,2,5-oxadiazol-3-amine	C_12_H_7_FN_8_O_2_	314.24
34	23.760	3.01	1,11-Diphenyl-1,3,5,7,9-undecapentaene	C_23_H_22_	298.4
35	23.849	2.29	3-Chloro-8-methylthio-11H-indolo[3,2-c] quinoline	C_16_H_11_ClN_2_S	298.8
36	24.000	4.01	1-Phenanthrenecarboxylic acid, 1,2,3,4,4a,4b,5,9,10,10a-decahydro-1,4a-dimethyl-7-(1-methylethyl)-, methyl ester, [1R-(1α,4aβ,4bα,10aα)]-	C_21_H_32_O_2_	316.48
37	24.290	2.83	N-(2,4-Dimethoxyphenyl)-6-fluoroquinolin-4-amine	C_17_H_15_FN_2_O_2_	298.31
38	24.378	0.72	Butanoic acid, 2-pentenyl ester, (Z)-	C_9_H_16_O_2_	156.22
39	24.592	0.82	Heptadecane	C_17_H_36_	240.5
40	25.235	14.54	2,3-Pyrazinediamine, N3-cyclopropyl-N2, N2-dimethyl-5,6-diphenyl	C_21_H_22_N_4_	330.4
41	25.386	1.87	1,4,10,13-Tetraazacyclooctadeca-4,9,13,18-tetraene-7,16-dione, 5, 9,14,18-tetramethyl-	C_18_H_28_N_4_O_2_	332.4
42	26.117	0.45	Heneicosane	C_21_H_44_	296.6
43	27.567	0.58	Tetracosane	C_24_H_50_	338.7

*Note:* Isolate active from: (a) I_nH_-1, (b) I_nH_-2, (c) I_EtOAc_-1, (d) I_EtOAc_-2, and (e) I_EtOAc_-3.

**Table 4:** Top 5 target genes of each active isolates related to cancer targets in the cancer pathway.

Source	Average shortest path length	Betweenness centrality	Closeness centrality	Clustering coefficient	Eccentricity	Edge count	Indegree	Name
Isolate active (I_nH-2_)—lung cancer	1.6	0.185198	0.625	0.323171	2	41	29	MMP2
	1.441176	0.475553	0.693878	0.288099	2	53	34	MDM2
	1.342105	0.720449	0.745098	0.235354	2	55	30	MAPK3
	0	0	0	0.249883	0	66	66	TP53
	1.142857	0.330877	0.875	0.249008	2	64	16	EGFR

Isolate active (I_EtOAc-1_)—breast cancer	1.625	0.092727	0.615385	0.326705	2	33	24	MMP2
	1.428571	0.887648	0.7	0.267633	3	46	29	MDM2
	1	0.343938	1	0.223835	1	58	56	STAT3
	1.111111	0.716947	0.9	0.225953	2	58	10	EGFR
	1.551724	0.430403	0.644444	0.32043	3	31	17	KIT

**Table 5:** Optimization and ADMET prediction of compounds from each active isolate.

Parameters	Active isolate (I_nH-2_)					Active isolate (I_EtOAc-1_)				
	1	2	3	4	5	1	2	3	4	5
Optimization										
Energy	−1026.78	−924.41	−1066.29	−1143.27	−992.14	−852.22	−1231.65	−927.95	−618.59	−1008.62
Moment dipole	2.75	1.31	1.99	9.62	3.60	2.05	0.50	2.24	1.60	2.12
Physicochemical										
MW (g/mol)	330.43	300.44	332.44	314.23	298.33	284.48	390.56	306.48	—	338.57
HBA	2	2	0	9	4	2	4	2	—	2
HBD	1	1	6	2	0	0	0	0	—	0
TPSA (Å^2^)	41.05	37.30	83.58	155.66	44.76	26.30	52.60	26.30	—	26.30
LogP	3.84	4.36	1.02	0.72	3.19	5.90	6.27	—	—	6.67
GI absorption	High	High	High	Low	High	High	High	—	—	Low
BBB permeant	Yes	Yes	No	No	Yes	No	No	—	—	No
Drug-Likeness	Yes	Yes	Yes	Yes	Yes	Yes	Yes	—	—	Yes
Toxicity prediction										
LD_50_ (mg/kg)	1000	1710	1970	1356	1000	5000	3200	20000	700	8000
Class	4	4	4	4	4	5	5	6	4	6

**Table 6:** Molecular docking results of active isolates with the cancer target protein MMP-2.

Lung cancer (PDB ID 3AYU)				Breast cancer (PDB ID 2D60)			
Compounds	Binding affinity (kcal/mol)	Binding constant (μM)	RMSD (Å)	Compounds	Binding affinity (kcal/mol)	Binding constant (μM)	RMSD (Å)
(b)1	−8.2	2.72	0.01	(c)1	−8.3	0.39	1.30
(b)2	−9.5	0.17	0.03	(c)2	−9.4	0.12	0.96
(b)3	−7.0	0.17	0.06	(c)3	−7.3	0.06	1.68
(b)4	−7.8	0.17	0.07	(c)4	−7.8	0.09	0.28
(b)5	−6.8	0.17	0.06	(c)5	−6.8	0.05	1.14
Native	−8.2	0.16	0.76	Native	−7.8	0.09	1.00
Doxorubicin	−7.7	0.17	0.07	Doxorubicin	−7.8	0.58	0.54

## Data Availability

The data that support the findings of this study are available from the corresponding author upon reasonable request.

## Supporting Information

Supporting information contains figures of the results of characterization of active isolates with TLC, figures of interpretation of functional groups with FT-IR (KBr), figures of interpretation of ^1^H and ^13^C-NMR, and superimposition of basic native ligand structures stating valid molecular docking results.
