# Supplementary material for: In Vitro Cytotoxic and Molecular Docking Studies of the Network Pharmacology Approach From Bioactive Compounds of Coleus amboinicus Leaves Against Lung and Breast Cancer Cells
Source: Adv Pharmacol Pharm Sci. 2025 Jul 11;2025:5946648. doi: 10.1155/adpp/5946648 (PMC12274097; doi:10.1155/adpp/5946648)
Supplement: Supporting Information — Additional supporting information can be found online in the Supporting Information section. [file 5946648.f1.docx]

***Supplement Information***

**In vitro cytotoxic and molecular docking studies of the network pharmacology approach from bioactive compounds of *Coleus amboinicus* leaves against lung and breast cancer cells**

Kasta Gurning^a,b^, Gian Primahana^c^, Endang Astuti^a^, Winarto Haryadi^a,*^

^a^Department of Chemistry, Faculty of Mathematics and Natural Sciences, Universitas Gadjah Mada, Yogyakarta 55281, Indonesia

^b^Department of Pharmacy, Sekolah Tinggi Ilmu Kesehatan Senior Medan, Medan 20141, Indonesia

^c^Research Center for Pharmaceutical Ingredients and Traditional Medicine, National Research and Innovation Agency (BRIN), South Tangerang 15314, Indonesia

Coressponding author: Prof. Dr. Winarto Haryadi; *e-*mail: wnrt_haryadi@ugm.ac.id


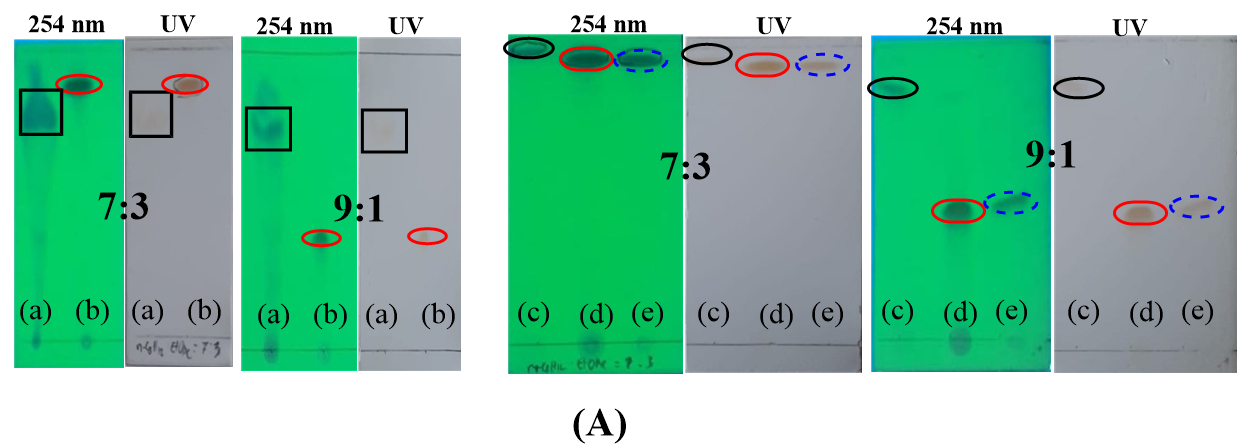


FIGURE S1. Characterization of active isolates using TLC with *n*-C_6_H_12_: EtOAc ratio eluent. Note (a) I_nH-1_, (b) I_nH-2_, (c) I_EtOAc-1_, (d) I_EtOAc-2_, and (e) I_EtOAc-3_.


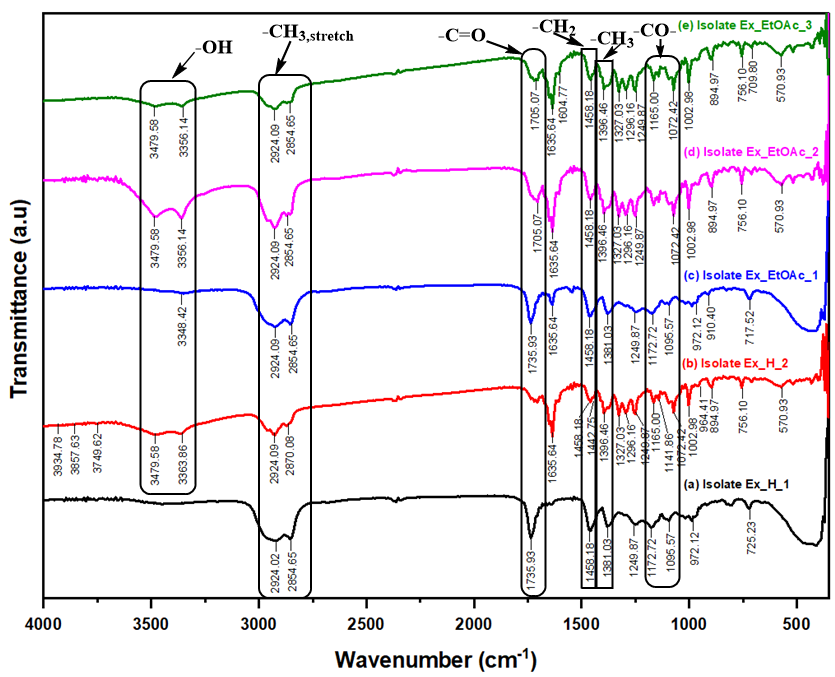


FIGURE S2: Identification of group absorption of each active isolate with FT-IR_(KBr)_: (a) I_nH-1_; (b) I_nH-2_; (c) I_EtOAc-1_; (d) I_EtOAc-2_; (e) I_EtOAc-3_.


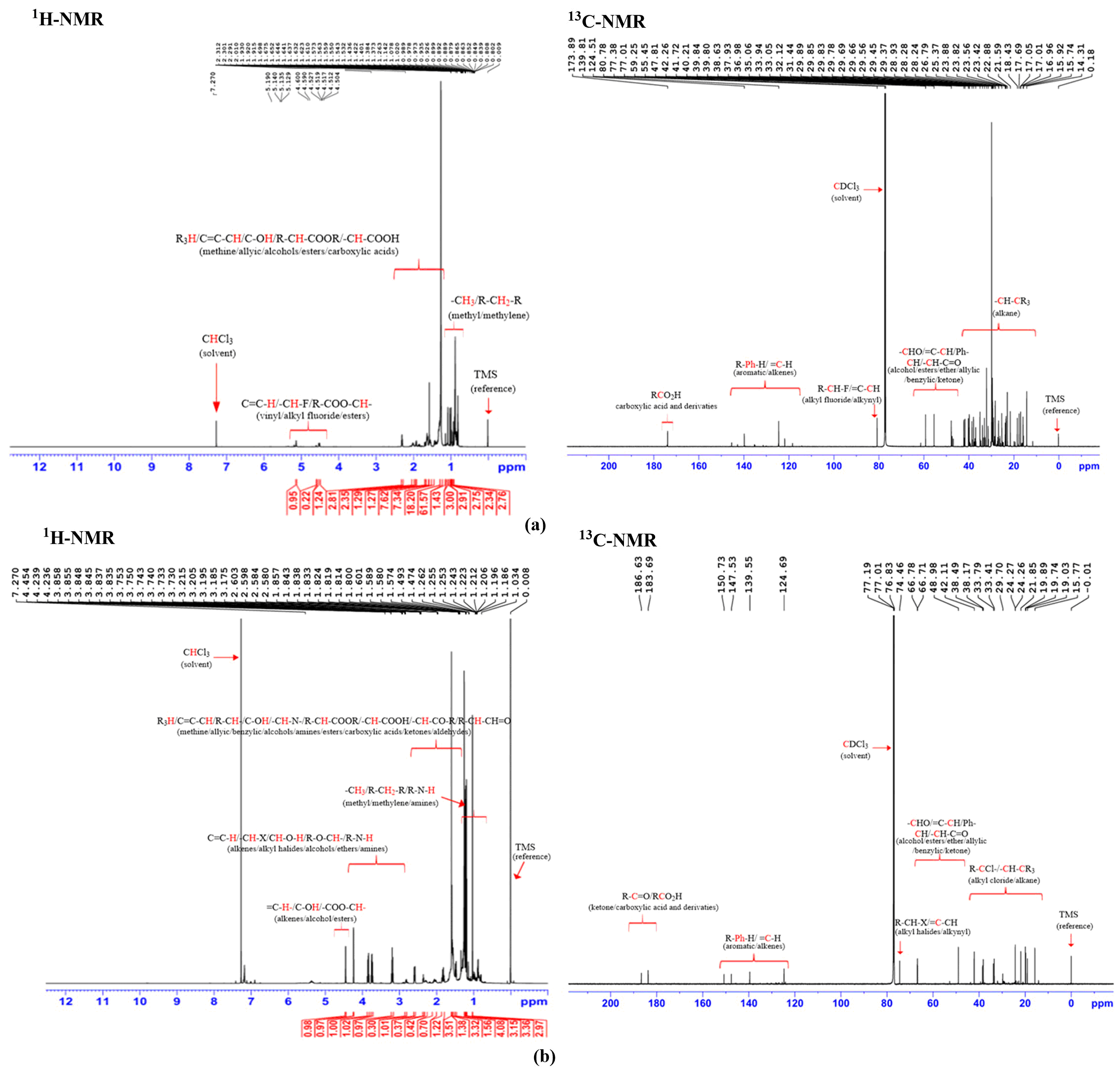


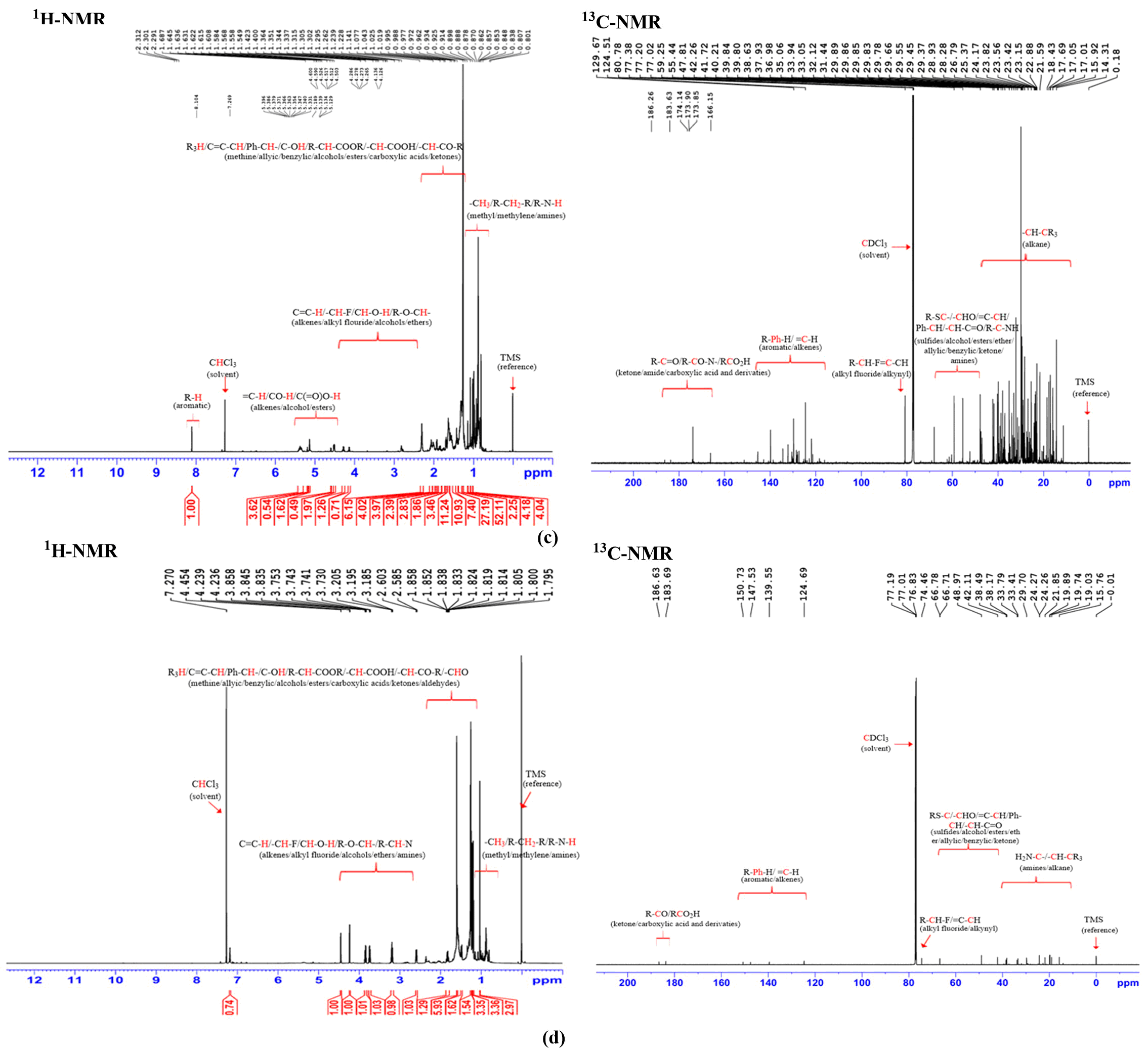


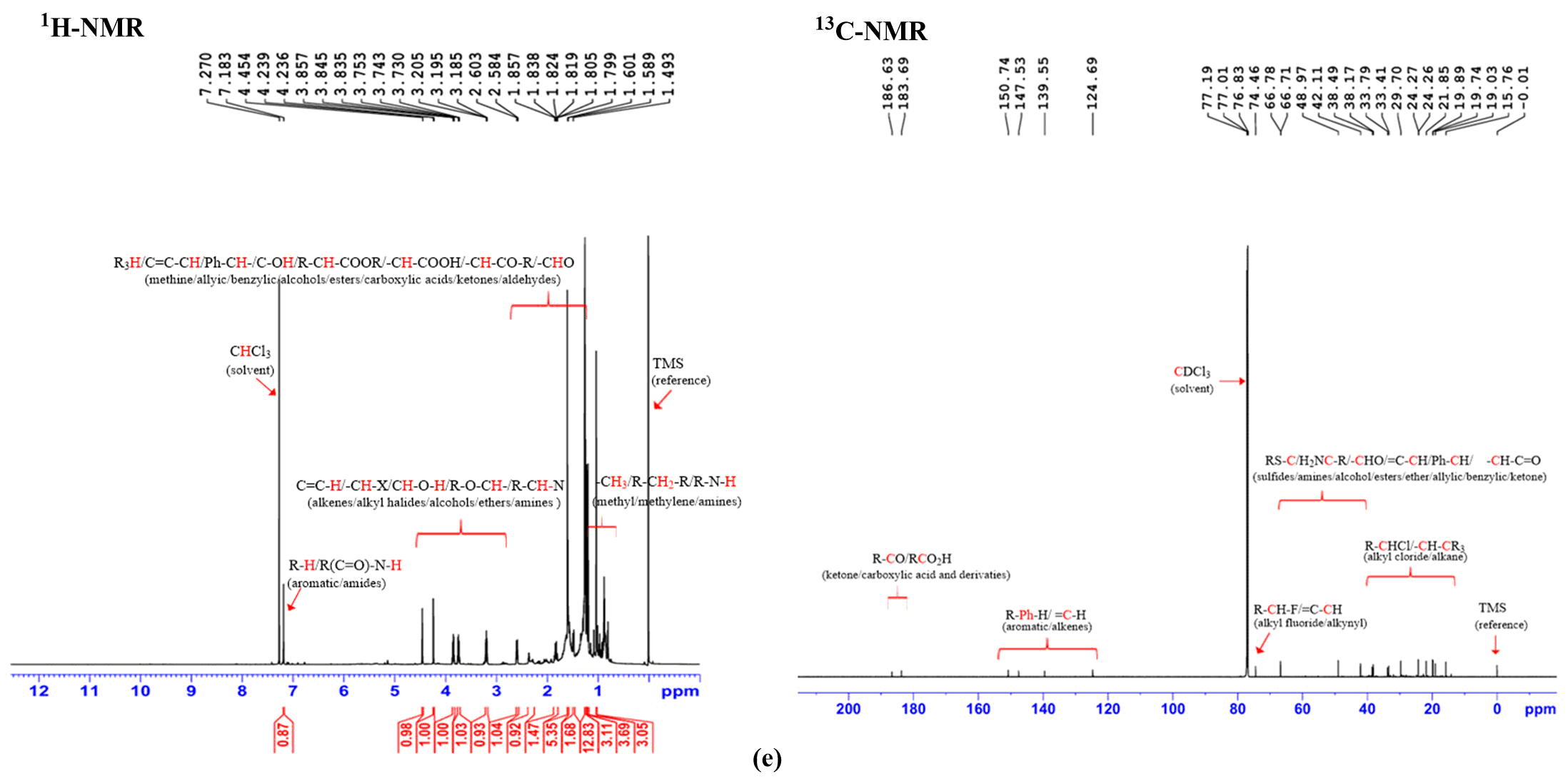


FIGURE S3: Analysis of the chemical structure of the isolate using ^1^H & ^13^C-NMR; (a) I_nH-1_; (b) I_nH-2_; (c) I_EtOAc-1_; (d) I_EtOAc-2_; (e) I_EtOAc-3_.


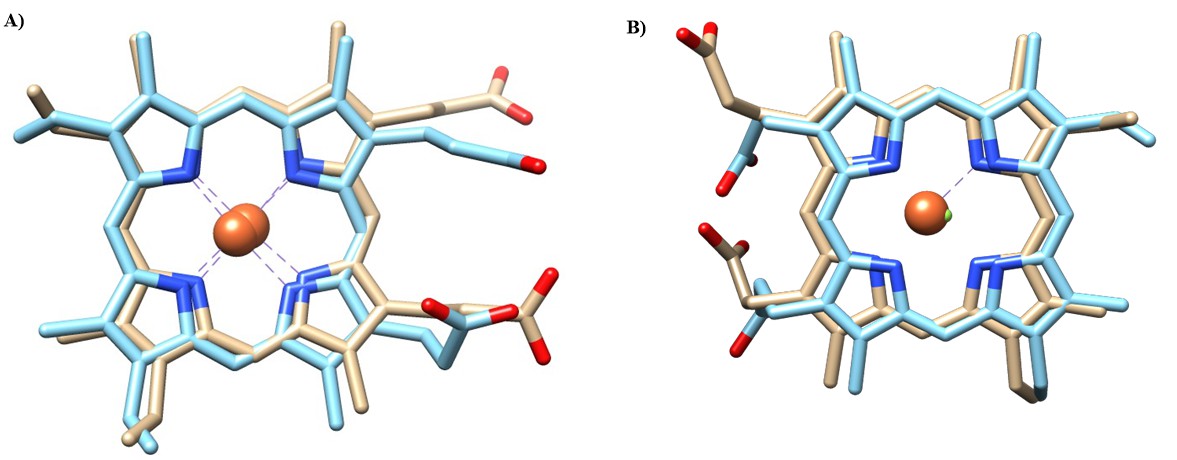


FIGURE S4. Superimposition structures of native ligands before (light blue) and after (light brown) the re-docking process in the active site of MMP-2, where the oxygen atoms are shown in red; (A) MMP-2 for lung cancer and (B) MMP-2 for breast cancer.
